# Evaluation of a digital reporting and supporting tool in breast cancer prevention trials (KarmApp)

**DOI:** 10.1186/s12913-025-12471-9

**Published:** 2025-03-06

**Authors:** José Tapia, Marike Gabrielson, Mattias Hammarström, Yvonne Wengström, Jenny Bergqvist, Aki Tuuliainen, Mikael Eriksson, Kamila Czene, Per Hall, Magnus Bäcklund

**Affiliations:** 1 Department of Medical Epidemiology and Biostatistics, Karolinska Institutet, Nobels Väg 12 A, Stockholm, 171 77 Sweden; 2 Department of Neurobiology, Care Science and Society, Division of Nursing and Theme Cancer, Karolinska CCC, Karolinska University Hospital, Stockholm, Sweden; 3 Department of Surgery and Oncology, Capio St Görans Hospital, Stockholm, Sweden; 4 Department of Oncology, Södersjukhuset, Stockholm, Sweden

**Keywords:** Adverse event reporting, Smartphone application, Randomized clinical trial, eHealth, mHealth, Breast cancer prevention, Anti-estrogen therapy

## Abstract

**Background:**

Anti-estrogens are widely used to reduce recurrence in breast cancer patients. The side effects often lead to treatment non-adherence and the use of anti-hormonal treatments as primary prevention in women with increased risk of breast cancer is very low. We have conducted breast cancer prevention trials aiming to lower the adverse effects of anti-hormones, but with retained effect. For increased two-way communication and to facilitate effective reporting of adverse events we have developed a smartphone application (app), the KarmApp. The aim of our study was to explore the user frequencies of the different features of the app, and if the use is influenced by age and has changed over time.

**Methods:**

Healthy women aged 40–74, attending the Swedish mammography screening program, were invited to participate in trials evaluating risk-reducing medications at different doses and formulations (KARISMA 2, *N* = 1,440, KARMA Creme, *N* = 90, and KARISMA Endoxifen, *N* = 240). After inclusion, participants were given instructions on how to use the app. We retrospectively evaluated the usage frequencies of the KarmApp and its various functions from 2016 to 2024. To explore the age factor attributed to KarmApp usage, age groups were formed and age was also analyzed as a continuous variable, using logistic regression.

**Results:**

Of 1,770 participants, 1,646 (93.0%) used the KarmApp and there were 17,065 user interactions, corresponding to 9.6 interactions per person. “Study Activities Overview” was the feature most frequently used. A total of 2,985 adverse events were reported, 2,309 (77.4%) via the KarmApp. The remaining reports were mainly done via phone calls. The younger age, the more likely women were to use the app (*p* < 0.001), but 75% of women in the highest age group, 60–74 years, used the app. The proportion of users increased over time.

**Conclusions:**

A vast majority chose to use the KarmApp and reported side effects via the app. More prevalent use was seen among younger participants and use increased over calendar period. Supported by our data, KarmApp exemplifies the potential of using mobile technologies in clinical trials.

## Background

Most breast cancer patients receive post-operative anti-estrogen therapy for 5–10 years. Anti-hormonal therapy is also used for breast cancer risk reduction in healthy women. However, in both settings adherence to these lengthy therapies is suboptimal due to the menopausal like side effects and, thus, a risk of poorer outcomes [1–3].

Today smartphones are widely used and smartphone applications (apps) are increasingly being utilized for self-care and health data tracking. Thus, their potential for improvement of treatment compliance is well recognized, but the introduction as a routine in cancer management has so far been modest, [4]. World Health Organization (WHO) defines telemedicine tools as “mHealth” or “eHealth,” which refers to “medical and public health practice supported by mobile devices, such as smartphones, patient monitoring equipment, personal digital assistants, and other wireless devices” [5].

So far health apps in oncology focus on patient support during adjuvant treatment, survivorship issues or rehabilitation [6, 7]. There are also knowledge gaps in how apps could empower clinical cancer trial participants and improve treatment adherence and adverse events (AE) reporting, in accordance with Good Clinical Practice (GCP) [8–10]. Likewise, there is a lack of information on how age influences the use [11]. Implementing apps in clinical trials may face various challenges, including development costs, legal issues, and negative attitudes among researchers, healthcare professionals, and study participants [9, 12].

During the last decade we have conducted several breast cancer-related randomized controlled trials (RCT) involving Swedish female healthy volunteers. In the studies we have tested potentially risk-reducing anti-estrogen medications at different doses and formulations [13–16]. In 2016 we developed a digital tool, the KarmApp, to support women participating in our three latest trials in being compliant to the study procedures [14–16]. In this paper we describe the app's content, development and usage patterns and present analyses on age and calendar period influence on its use.

## Methods

The aim of our study was to explore the user frequencies of the different features of the KarmApp, and if the use is influenced by age and has changed over time. From the data generated in our interventional trials potential differences over time and across age groups, in relation to other routes of two-way communication between participants and study staff, were explored. The study population consists of women aged 40–74 who participated in the Swedish mammography screening program. Women invited for a mammography screen also got an invitation to the three breast cancer prevention studies; KARISMA 2 [14], KARMA Creme (ATOS-010) [15] and KARISMA Endoxifen (ATOS-016R) [16]. Our study was designed as a post hoc retrospective evaluation of the KarmApp, mainly with descriptive data on the development process, usage frequencies, taking age and changes over calendar period into consideration.

KarmApp is a smartphone app developed within our academic research group [17] at the Department of Medical Epidemiology and Biostatistics, Karolinska Institutet, Stockholm, Sweden, for the interventional trials in the breast cancer project known as KARMA (KARolinska Mammography study for Risk Prediction of Breast Cancer) [18]. KarmApp was accessible on Android and iOS platforms. It is a Swedish app and is not commercially available. It is specifically designed for our clinical trials, although built as a generic tool with the potential to be adapted to other studies. It was developed with the primary purpose of facilitating two-way communication between the study staff and participants.

Here we in detail describe the development, functionality and emerging versions of KarmApp:

### KarmApp general architecture

**Fig. 1:**
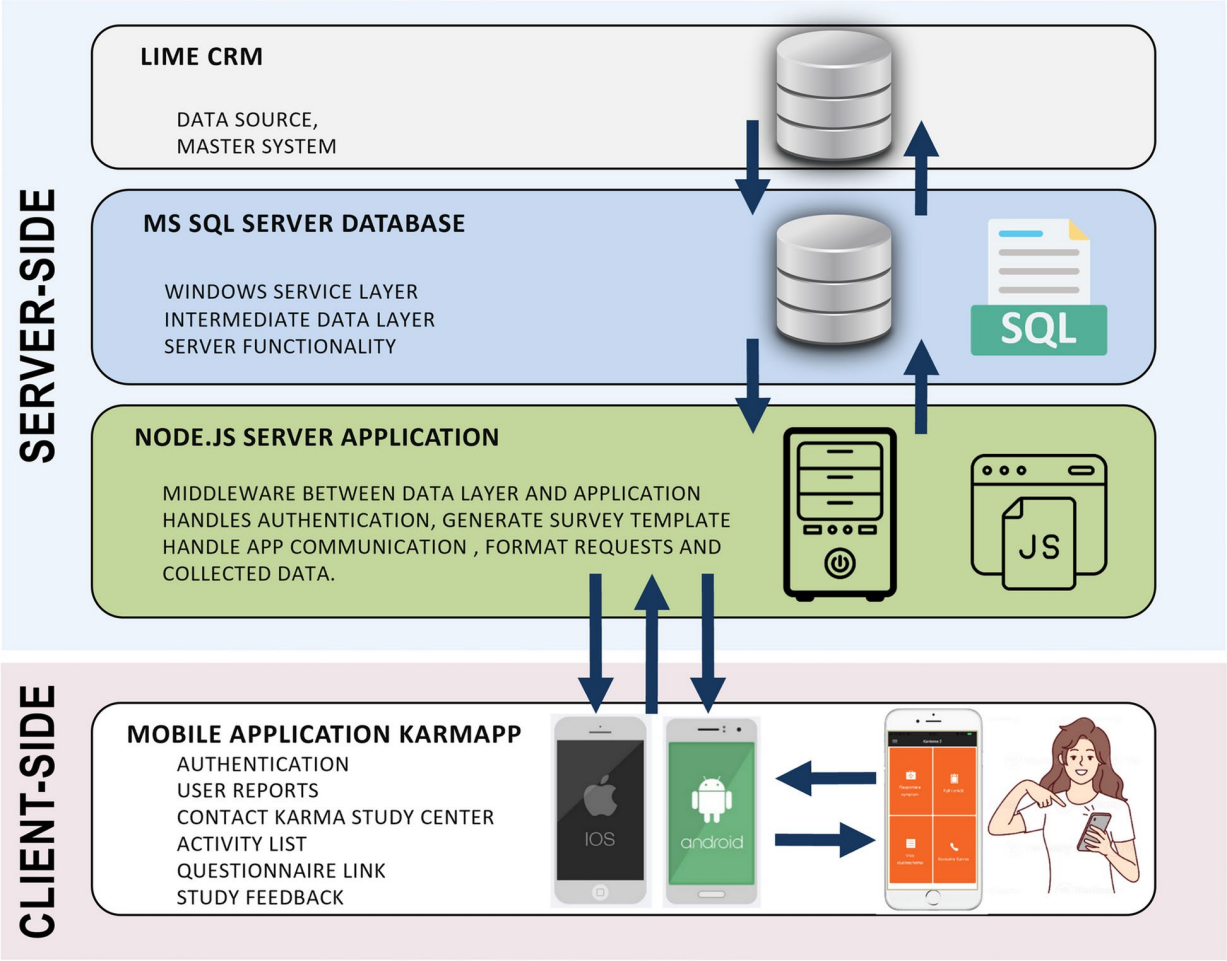
KarmApp client–server architecture. The KarmApp platform uses a client–server model with a client app for iPhones and Android smartphones. It employs Node.js as the server application, which handles communication with a Microsoft SQL Server database through application programming interfaces, ensuring data transmission and retrieval

The KarmApp platform follows a client–server model architecture [19], comprising a client application that utilizes services provided by a server application (Fig. 1). The client side of the app is designed to be compatible with iPhones and Android smartphones, serving as the user interface for interacting with KarmApp's features. For the server application, Node.js, a JavaScript runtime [20] was employed. The Node.js application functions as the background server, facilitating communication between the client side and the underlying infrastructure. It interacts with a Microsoft SQL Server database [21] for data storage. Application Programming Interfaces (APIs) were developed to manage the app's functionality, enabling data transmission to, and retrieval from, the database. All logins, messaging, and other communication are encrypted to ensure secure data transfer and maintain privacy. We use HTTPS, which employs the Transport Layer Security (TLS) protocol– formerly Secure Sockets Layer (SSL)– to encrypt communications.

KarmApp is designed to allow integration with other systems and to secure data transfer. The Customer Relationship Management (CRM) systems used for the respective trial was for KARISMA 2 and KARMA Creme, Microsoft Dynamics CRM [22] and for KARISMA Endoxifen, LIME CRM [23]. They both have Case Report Forms (CRF) as integrated parts. For example, when an AE was reported it automatically appeared in the CRF, enabling an evaluation by a trial physician.

The KarmApp's interface elements, including pages, fields, forms, icons, text, buttons, labels, and more, are controlled through the database and server. This minimizes the need for frequent app releases for minor interface changes, eliminating the requirement for users to frequently update the app. As part of KarmApp, an integrated automated system can send text messages as Short Message Services (SMS) to the study participants, serving as reminders for various activities during their participation.

**Fig. 2:**
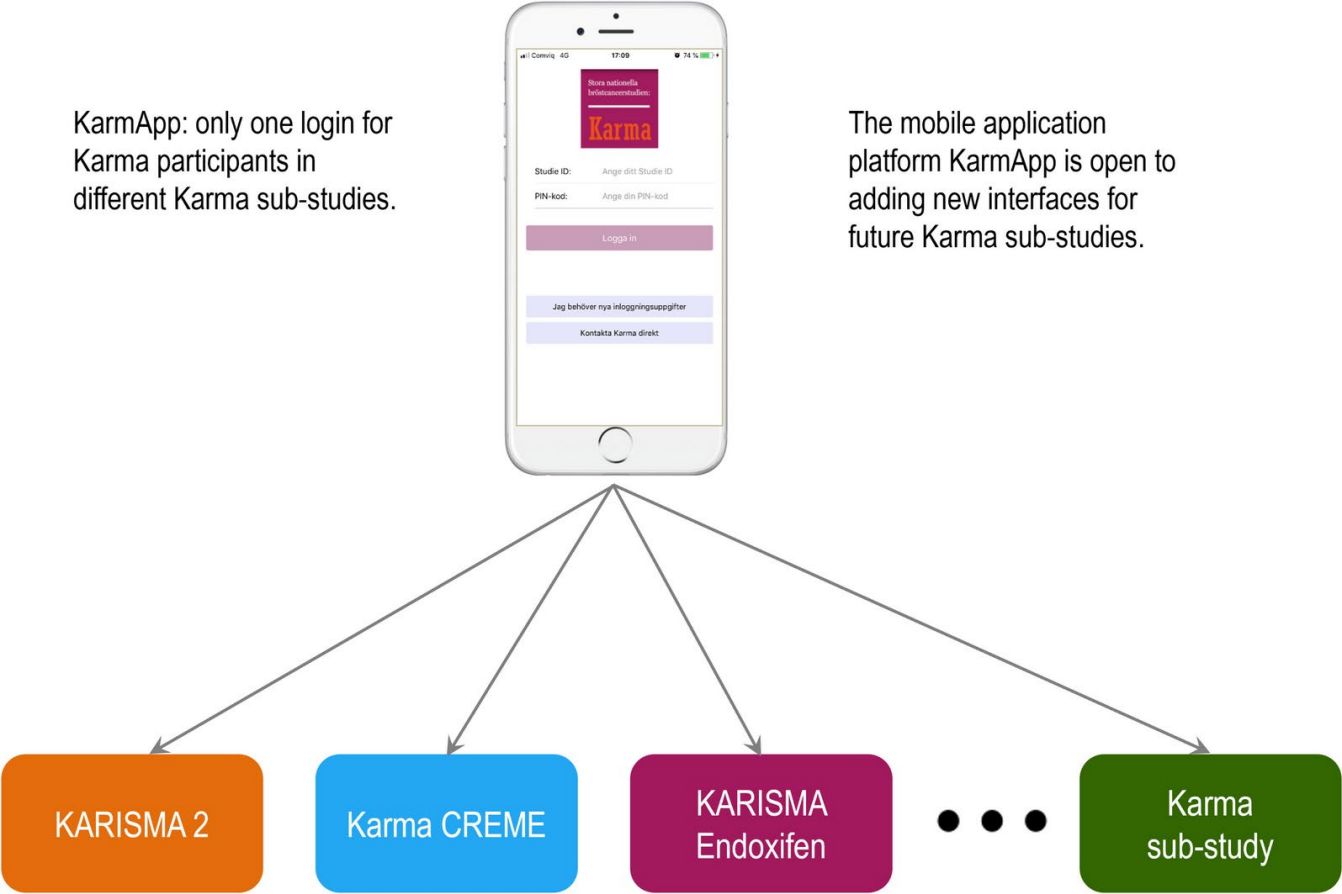
The figure illustrates the current interfaces within the KarmApp platform for present and future Karma sub-studies. KarmApp offers simple user experience by a unified login system

Furthermore, KarmApp has been designed as a generic platform capable of accommodating new interfaces for future KARMA sub-studies. The initial interface, developed for the KARISMA 2 study (version 1 and 2), was later redesigned with additional interfaces for the KARMA Creme study (version 3) and KARISMA Endoxifen (version 4) (Fig. 2).

### KarmApp features

**Fig. 3:**
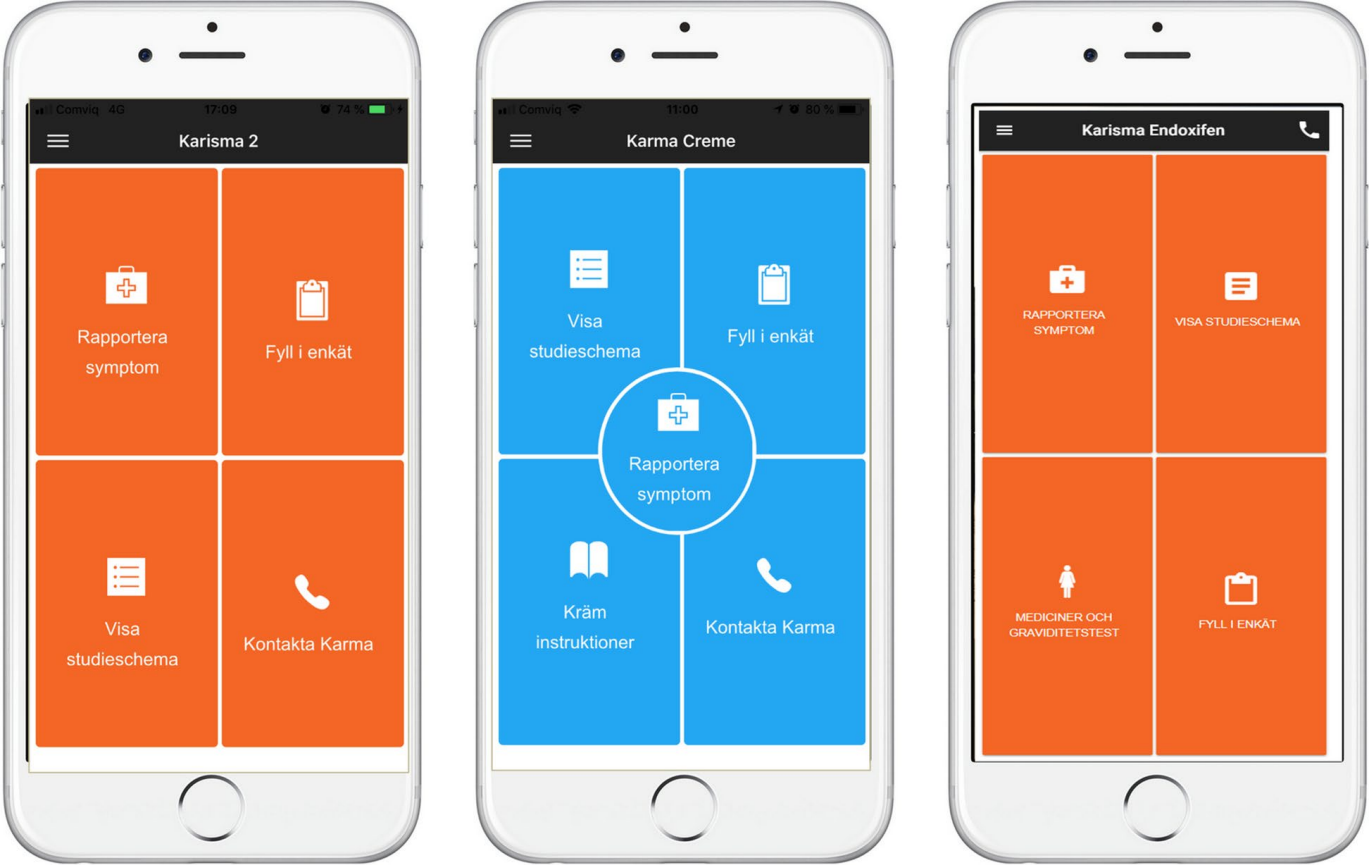
Pictures of the start pages shown when the study participant opens KarmApp, visualized for each study. For KARISMA 2, to the left (translated from Swedish: Report symptoms / Fill in questionnaire / Show study schedule / Contact Karma); for KARMA Creme, in the middle (translated from Swedish: Show study schedule / Fill in questionnaire / Report symptoms / Creme instructions / Contact Karma) and to the right for KARISMA Endoxifen (translated from Swedish: Report symptoms / Show study schedule / Medications and Pregnancy tests / Fill in questionnaire)

KarmApp offers a range of features that facilitate the study participation (Fig. 3).

#### Adverse events reporting

KarmApp enables participants to report AE spontaneously and continuously during the whole trial period. Symptoms can be entered as free text or be selected from predefined lists. Participants can indicate the severity of symptoms, according to international GCP standards [24] and provide start and stop dates if required by the study protocol. Typical AE in KARISMA 2 [25] and KARISMA Endoxifen (preliminary data, manuscript in preparation) were menopausal-like symptoms as hot flashes and night sweats and in KARMA Creme, in which the anti-estrogen was topically applied, skin rashes [15]. AE Reporting of potential side effects can also be done via various other sources such as telephone, email, or during a visit to the study center.

#### Personalized study activities overview

Participants can access an overview of their study activities, dates for their visits and task completion status. The feature provides a comprehensive view of the different activities associated with their study participation.

#### Access to study questionnaires

KarmApp provides an embedded link to the web-based study questionnaires, allowing participants to complete them using the app, instead of a computer.

#### Contact with study staff

KarmApp enables direct communication between participants and study staff. Users can initiate phone calls, send text messages, or access the study webpage, facilitating communication and support.

#### Reminders for study medications

Participants can configure reminders within the app to support a timely administration of study medications. By this function push notifications can serve as reminders for users to take their study medications.

#### Pregnancy test report

If applicable, participants can report their home-based pregnancy test results through KarmApp.

#### Ongoing medication report

Participants can report any changes in their concomitant medication and indicate if they were non-compliant with the study medication.

#### Post-study feedback

**Fig. 4:**
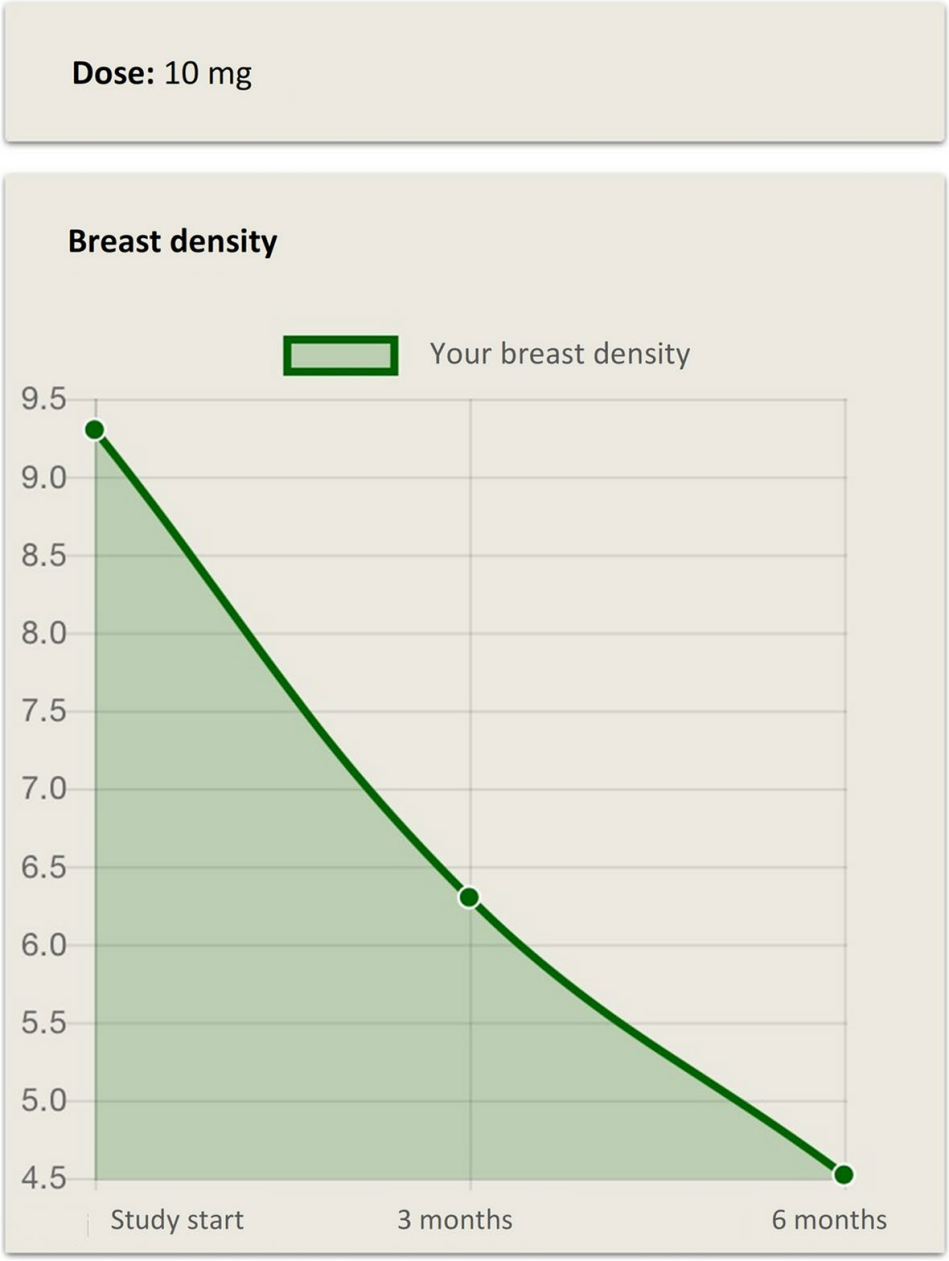
Example of post-study feedback. After study’s completion participants can view personalized information. The figure shows an example of post-study feedback including details on assigned dose and change in mammographic breast density over time

After the study’s database lock for data analysis, the participants are offered feedback on study results allowing them to view personalized information about their participation. This may include details such as the assigned dose or other relevant study-related information (Fig. 4).

### Development of KarmApp

In general, app development involves several stages and follows a systematic and iterative approach [26]. For use as eHealth tools questions regarding security, privacy and human–computer interaction must be answered to protect sensitive patient information.

Specifically, KarmApp intends to meet three key user preferences for mobile technology in clinical trials [12]:

- *Instant support*: Participants should be able to contact the study center with questions, including technical issues, minimizing the need to contact the device manufacturer or app developer.
- *Secure data*: Ensuring secure data transfer and storage, by using a database server behind fire walls at the Department of Medical Epidemiology and Biostatistics at Karolinska Institute. Participant data is accessible only to authorized Karma personnel.
- *User-friendly*: A user-friendly interface, prioritizing ease of use and intuitiveness. The app should require minimal navigation training and be easy to learn, to ensure o low threshold for its use.

The following features and requirements were also considered during the initial development and further on:

#### Requirements specification

The requirements for KarmApp were defined based on the study protocols through extensive consultation with the study staff. A collaborative effort was undertaken to gather a comprehensive list of requirements outlining functionality and features of the app.

#### Design

Considering the defined requirements, the development team created a detailed design and architecture for KarmApp. This included wireframes and user flow diagrams to visualize the structure and interface. Visual identity was defined through color schemes, typography, and icons to thriving for a consistent and appealing user experience (Fig. 3).

#### Implementation

App functionality was implemented using Angular programming language in the Ionic framework, SQL-server programming scripts, JavaScript on the Node.js runtime, and platform-specific tools for iOS and Android. Regular testing and debugging were conducted throughout the implementation phase to identify and address software defects or usability issues.

#### Testing

KarmApp underwent comprehensive testing to ensure the required functionality and user-friendliness. The testers followed a structured test protocol aligned with the user requirement specifications for user testing, systematically evaluating the app's performance against predefined criteria. The app's development was guided by a test-driven development (TDD) approach, a methodology known to be supportive in achieving robust and reliable functionality by writing test cases before coding. The test cases were designed and executed based on the functional requirement specifications, to verify that all implemented features met the intended functional criteria.

#### Deployment

KarmApp was deployed to the Apple App Store [27] and Google Play Store [28], making it available to designated end users. This stage involved preparations and processes to release the app to the intended users, including submission, review, and approval by app store administrators.

#### Post-release maintenance

After KarmApp was released, continuous monitoring mechanisms were implemented to track its performance and identify areas for improvement. A change log was maintained to document reported bugs and potential enhancements. Each entry in the change log was assessed for risk level, guiding the prioritization and safe implementation of changes. This systematic approach ensured that updates and bug fixes enhancing functionality and user experience were released efficiently.

### KarmApp versions

#### Version 1

The initial version of KarmApp (v. 1.3.0), launched in November 2016, focused on key features such as AE reporting, online questionnaires, participant activity overview, integration with an automated text message system, and direct communication with study staff. In 2017, during its initial development phase, KarmApp underwent an end-user evaluation early during the KARISMA 2 trial. Out of 34 participants contacted, 22 women participated in structured telephone interviews, providing feedback on user experiences and user-friendliness. The interviews revealed that 17 out of 22 individuals had downloaded KarmApp, primarily for questionnaires. However, only some discovered the study schedule feature due to insufficient information. Thus, we onwards included that in the user manual. Users also encountered technical issues, with one in three facing login difficulties. Another important finding was that respondents could not provide free-text responses to questions. This evaluation, alongside Patient-Reported Experience Measures [29], contributed to further app development and the creation of a user guide for KarmApp.

#### Version 2

The second version (v. 1.4.3), launched later in 2017, introduced significant upgrades involving a complete change in source code and interface design. The focus was on improving user-friendliness and performance based on the input from the evaluation.

#### Version 3

In the third version (v. 1.5.1), the app's architecture was redesigned to accommodate an additional interface for the KARMA Creme study. From this version the flexible inclusion of new interfaces for future sub-studies was also included. The post-study feature "Feedback to participants" was introduced, but remained disabled for participants until the completion of the trials. Potential feedback information is the assigned dose and individual and grouped results of change in breast density, frequencies of AE and quality of life estimates.

#### Version 4

The fourth version of KarmApp (v. 1.6.4), launched in November 2021. This version added an interface for KARISMA Endoxifen that includes new features for reporting pregnancy test results and changes in concomitant medications. The database structure and model underwent a redesign, incorporating changes such as adding new tables, modifying existing relationships, optimizing performance, and enhancing scalability, and the integration with the CRM system LIME was implemented.

#### Version 5

The current version of KarmApp (v. 1.7.0) launched in March 2024, includes the interface for our next Karma sub-study Stockholm MAmmography Risk stratified Trial (SMART) [30].

As mentioned above, under Post-Release Maintenance, we continuously made improvements based on feedback from both participants and study staff.

### Study population

Potential participants for our breast cancer prevention RCTs were invited before visiting the Breast Centre for their routine mammography screening, which in Sweden biannually targets women 40–74 years. If interested after reading the written study information they were orally informed by a trial physician and included after giving written informed consent. KARISMA 2 recruited participants from November 2016 to March 2019 (*N* = 1,440) [14], KARMA Creme from August 2018 to October 2018 (*N* = 90) [15] and KARISMA Endoxifen from December 2021 to November 2023 (*N* = 240), with the last data öpoints collected in June 2024 [16]. During the inclusion visit participants received a text message containing the login details and instructions for downloading and installing KarmApp on their mobile devices. Additionally, the study staff provided participants with a user guide with orientation to all sections of the app.

### Data analysis

Frequency numbers were produced for any usage of KarmApp, defined as having logged in to the app at least once. The KarmApp features were categorized into study activities overview, feedback to participants, AE reporting, request a new login code and configure reminders. User interactions with the app's features were tracked through a log-tracking function integrated into the app's functionality, recording all interactions in a centralized log table for detailed usage analysis.

To evaluate the frequencies and sources of reported AE, we used data reported via KarmApp and other sources, all documented in the CRF system. We also calculated the frequencies of use for the different weekdays and different hours during the day.

To explore the age factor attributed to KarmApp usage, age at study inclusion was categorized into three age groups 40–49, 50–59 and 60–74 years. Since KARISMA Endoxifen was only open for women ≤ 55, we performed additional, separate analyses of women aged 40–55. We also analyzed age as a continuous variable and its potential impact on KarmApp usage frequencies.

The number of reported AE was categorized into 1, 2, 3, 4, 5 and > 5 times and the reporting source was categorized into app, phone, email, visit to the study center and SMS.

We utilized exploratory data analysis with descriptive statistics and visualizations, providing graphical representations to identify patterns, trends and potential outliers. When age was analyzed as a continuous variable logistic regression was used. Analyses were conducted using SAS version 9.4 and R version 4.2.1.

### Ethical permissions

When the participants gave written informed consent to participate in the interventional trials, they consented to the handling of their data. All three trials were approved by the regional ethics board in Stockholm (for KARISMA 2 with the registration number 2016/651–31/2, for KARMA Creme with 2018/402–31 and for KARISMA Endoxifen with 2021/037–57).

## Results

A total of 1,770 women participated in the three RCT KARISMA 2, KARMA Creme and KARISMA Endoxifen, thus had the opportunity to use the KarmApp. The proportion of participants using any of the KarmApp features were 91.9%, 91.1% and 100%, respectively (Table 1). Descriptive statistics, categorized into three age groups, are presented in Table 2. Among the participants, 1,646 out of 1,770 (93.0%) used KarmApp, with the highest frequency of usage observed in the two younger age groups. Since KARISMA Endoxifen was only open for women ≤ 55, we performed a separate analysis for the group aged 40–55. The proportion of participants ≤ 55 using any of the KarmApp features in the three studies were 96.5%, 97.9% and 100%, respectively (Table 3).

**Table 1:** Proportion of participants using KarmApp, divided by sub-study. Numbers in parentheses represent the proportions (in %) specific to each category

Sub-study	Yes (%)	No (%)	Total
KARISMA 2^*^	1324 (91.9)	116 (8.1)	1440
KARMA Creme^**^	82 (91.1)	8 (8.9)	90
KARISMA Endoxifen^***^	240 (100.0)	0 (0.0)	240
	**1646 (93.0)**	**124 (7.0)**	**1770**

^*^Recruited participants November 2016 to March 2019

^**^Recruited participants August 2018 to October 2018

^***^Recruited participants December 2021 to November 2023

**Table 2:** Proportion of participants who used KarmApp, divided by age group. Numbers in parentheses represent the proportions (in %) specific to each category

Age Group	Yes (%)	No (%)	Total
**40– 49**	695 (98.7)	9 (1.3)	704
**50– 59**	510 (94.4)	30 (5.6)	540
**60– 74**	441 (83.8)	85 (16.2)	526
	**1646 (93.0)**	**124 (7.0)**	**1770**

**Table 3:** Proportion of participants (age ≤ 55) using KarmApp, divided by sub-study. Numbers in parentheses represent the proportions (in %) specific to each category

Sub-study	Yes (%)	No (%)	Total
**KARISMA 2**	762 (96.5)	28 (3.5)	790
**KARMA Creme**	46 (97.9)	1 (2.1)	47
**KARISMA Endoxifen**	240 (100.0)	0 (0.0)	240
	**1048 (97.3)**	**29 (2.7)**	**1077**

**Fig. 5:**
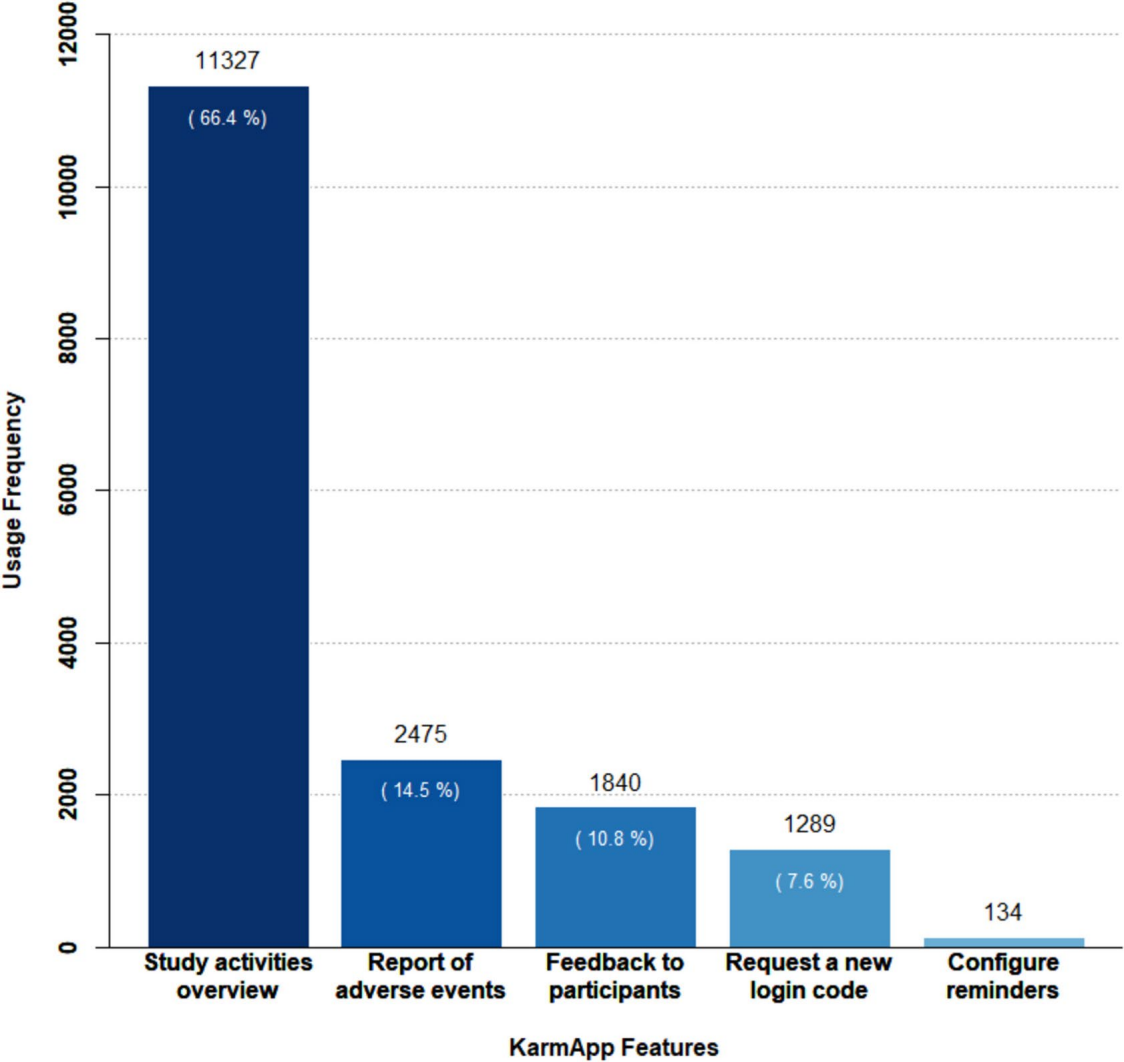
Frequencies of KarmApp feature usage. Diagram plotting the frequencies of user interactions with the different KarmApp features (total *N* = 17,065). Numbers in parentheses represent the proportions (in %) specific to each category

There were 17,065 user interactions with KarmApp, corresponding to 9.6 interactions per individual participant. The most frequently used feature, "Study Activities Overview" was accessed 11,327 times (66.4%). The "Report of Adverse Events" feature was used 2,475 times (14.5%). Furthermore, the "Feedback to Participants" feature was used 1,840 times (10.8%) (Fig. 5).

**Fig. 6:**
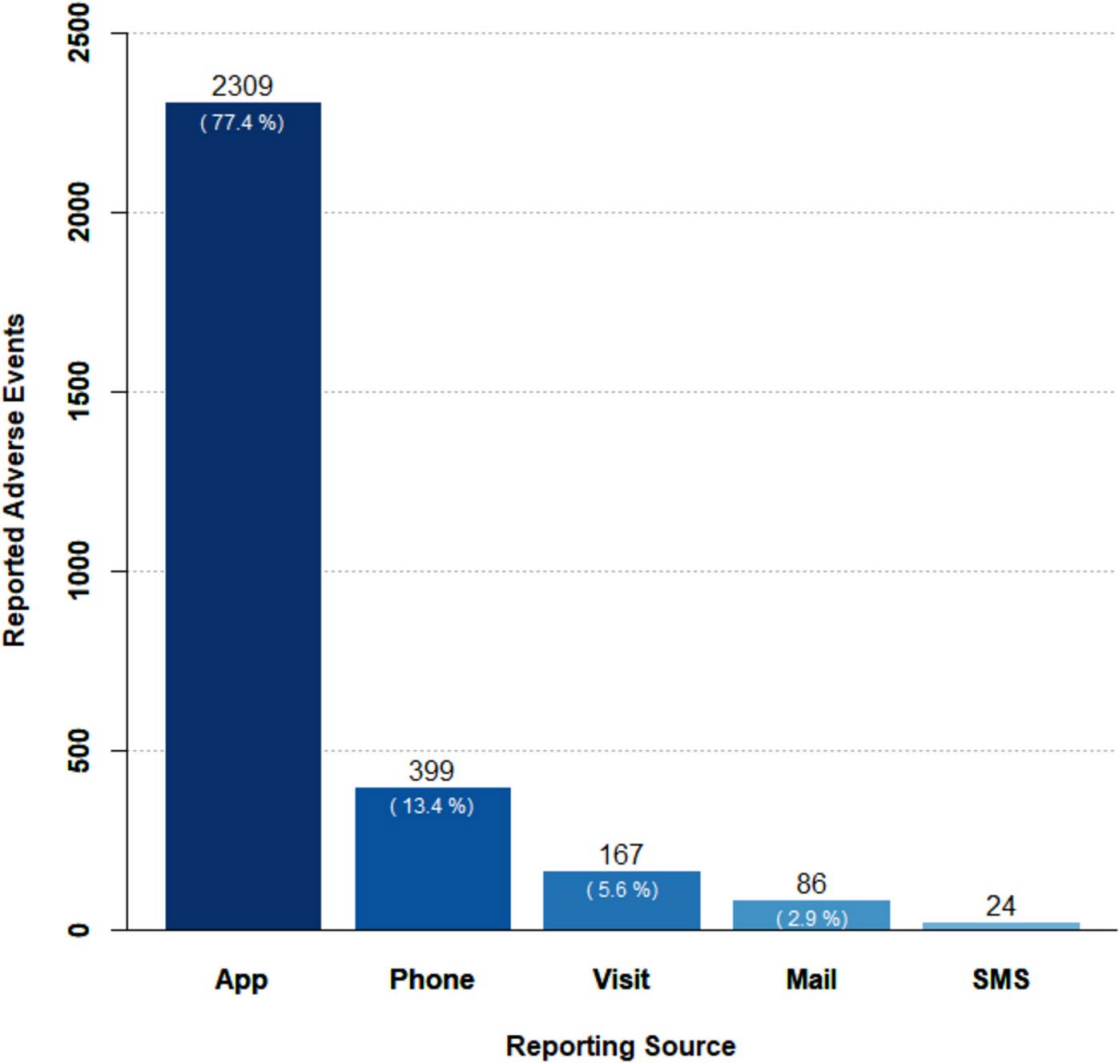
Diagram plotting the frequencies of reported adverse events by reporting source. Numbers in parentheses represent the proportions specific to each category

A total of 2,985 spontaneous AE reports were received from various sources. The majority, 2,309 (77.4%), were reported through KarmApp, followed by phone calls (399 or 13.4%). Other reporting sources accounted for less than 9% (Fig. 6).

The proportion of participants reporting AE seemed to increase from KARISMA 2 (31.0%), starting recruitment in 2016, to KARMA Creme (47.8%), starting recruitment in 2018, to KARISMA Endoxifen (77.1%), starting recruitment in 2021 (Table 4). When analyzing the participants across three age groups, the highest frequency of KarmApp usage for AE reporting was observed in the two younger age groups (Table 5).

**Table 4:** Proportion of participants reporting adverse events using KarmApp, divided by sub-study. Numbers in parentheses represent the proportions (in %) specific to each category

Study	Yes (%)	No (%)	Total
KARISMA 2^*^	447 (31.0)	993 (69.0)	1440
KARMA Creme^**^	43 (47.8)	47 (52.2)	90
KARISMA Endoxifen^***^	185 (77.1)	55 (22.9)	240
	**675 (38.1)**	**1095 (61.9)**	**1770**

^*^Recruited participants November 2016 to March 2019

^**^Recruited participants August 2018 to October 2018

^***^Recruited participants December 2021 to November 2023

**Table 5:** Proportion of participants reporting adverse events using KarmApp, divided by age group. Numbers in parentheses represent the proportions (in %) specific to each category

Age Group	Yes (%)	No (%)	Total
40– 49	336 (47.7)	368 (52.3)	704
50– 59	216 (40.0)	324 (60.0)	540
60– 74	123 (23.4)	403 (76.6)	526
	**675 (38.1)**	**1095 (61.9)**	**1770**

**Table 6:** Proportion of participants (age ≤ 55) reporting adverse events using KarmApp, divided by sub-study. Numbers in parentheses represent the proportions (in %) specific to each category

Study	Yes (%)	No (%)	Total
KARISMA 2	282 (35.7)	508 (64.3)	790
KARMA Creme	26 (55.3)	21 (44.7)	47
KARISMA Endoxifen	185 (77.1)	55 (22.9)	240
	**493 (45.8)**	**584 (54.2)**	**1077**

**Fig. 7:**
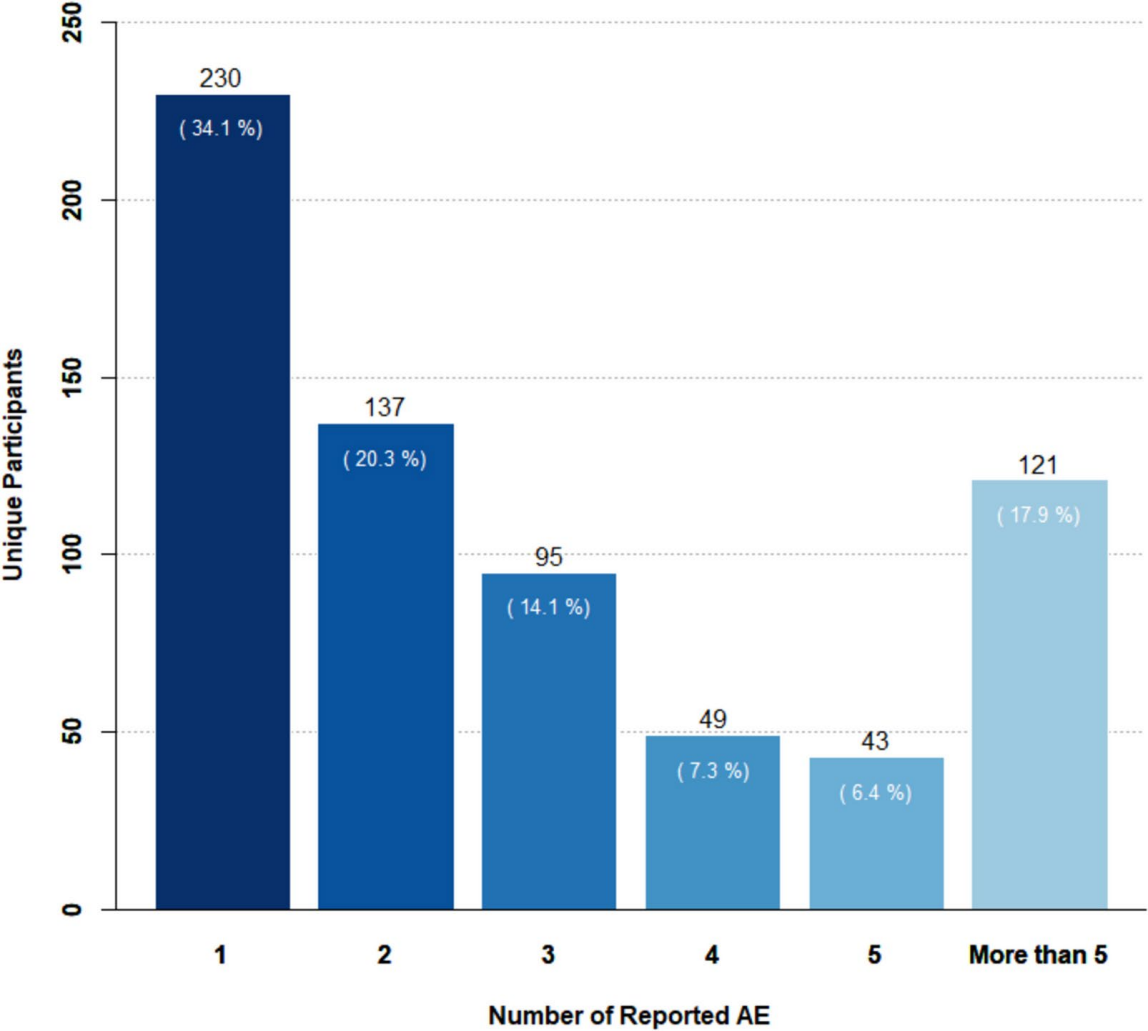
Diagram plotting the frequencies of adverse events reported by unique participants. The numbers along the horizontal scale indicate how many adverse events each unique participant reported during the study period. Numbers in parentheses represent the proportions specific to each category

Out of the 1,770 participants 675 (38.1%) reported at least one AE via KarmApp during the study period. Among these 675, 230 (34.1%) AE reported once, 137 (20.3%) twice and 121 participants (17.9%) more than five times (Fig. 7).

**Fig. 8:**
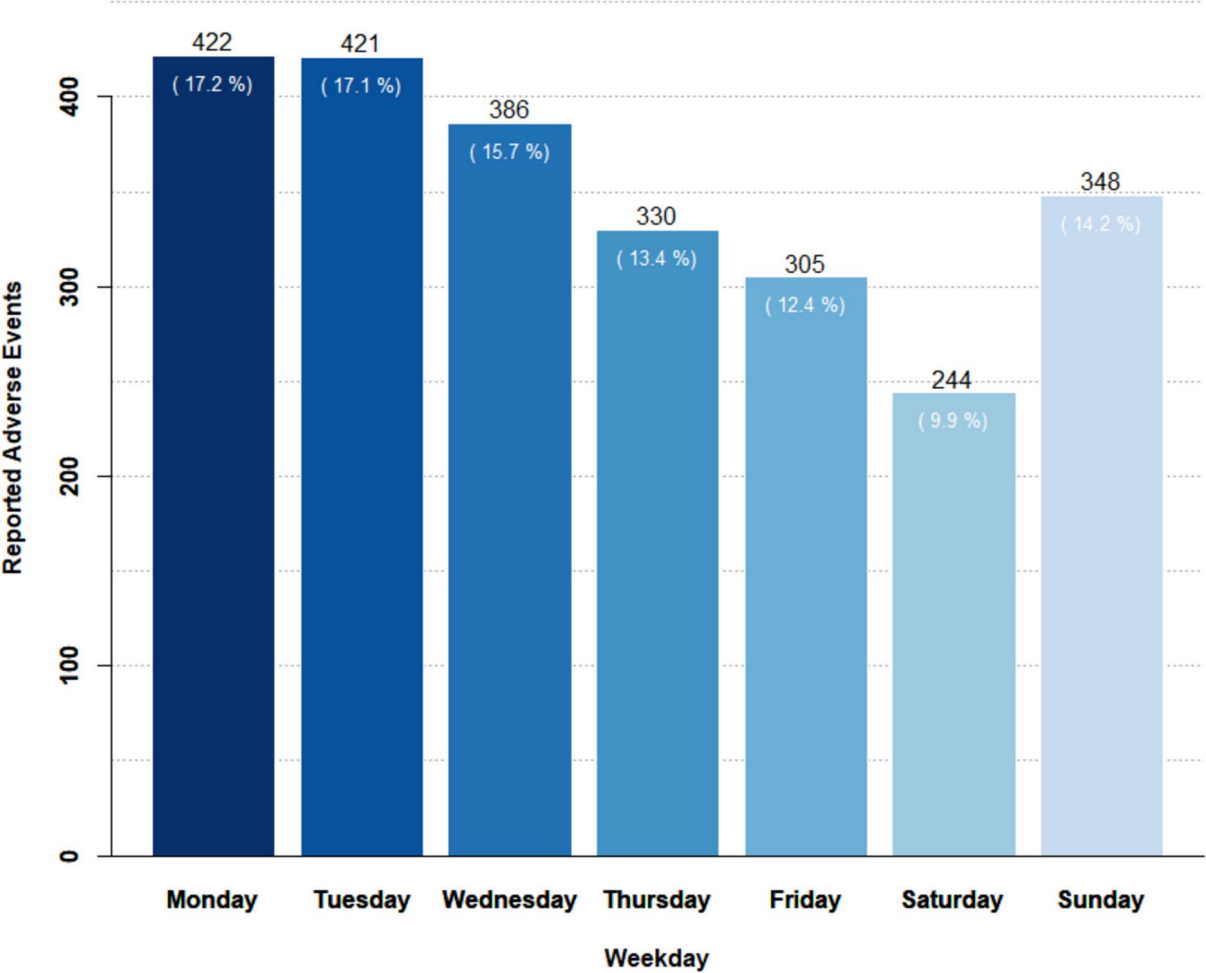
The figure presents the reported adverse events distribution based on the corresponding weekdays

**Fig. 9:**
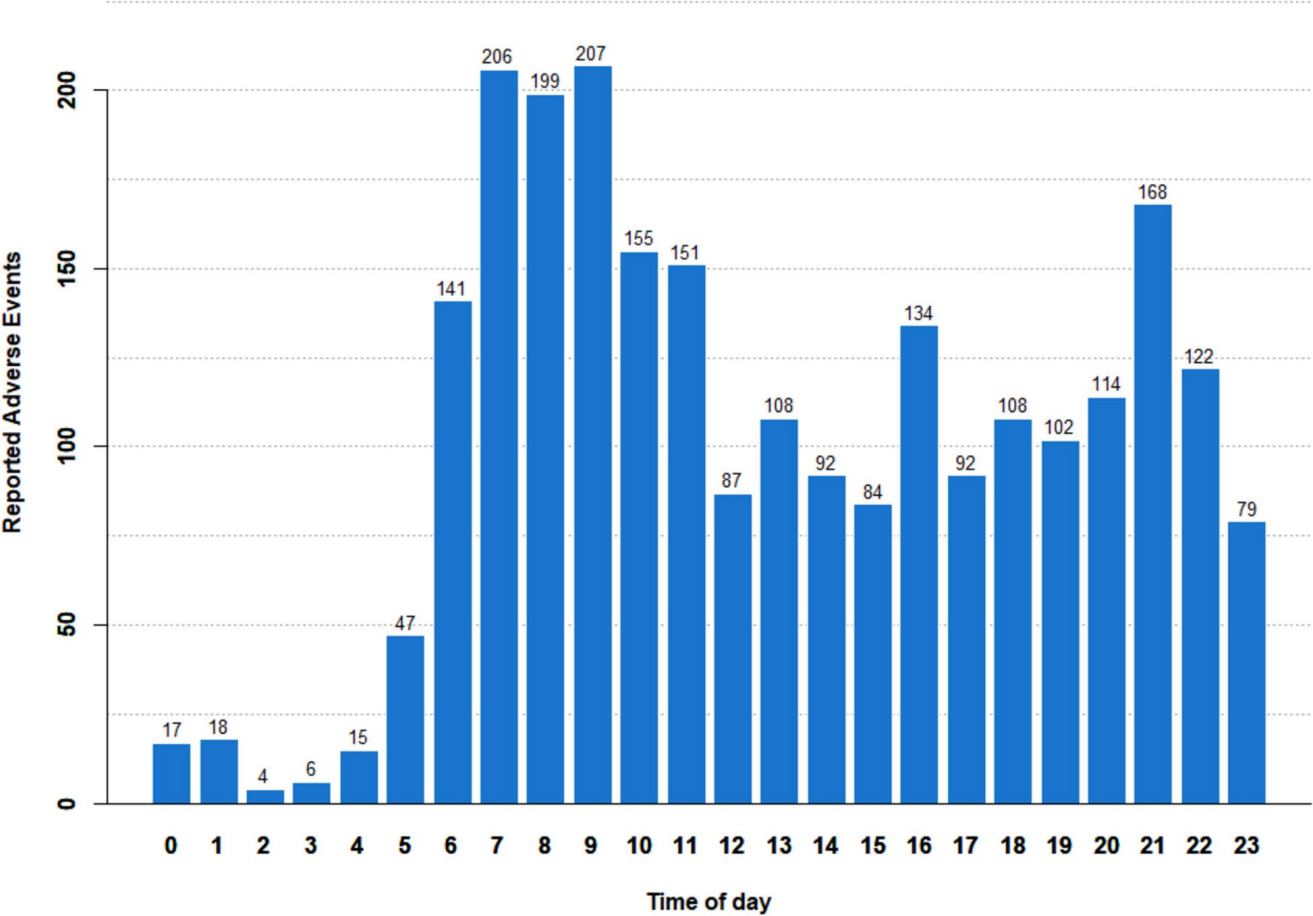
The figure depicts the frequency of reported adverse events based on the time of day

Monday and Tuesday were the weekdays with the highest frequency of AE reporting, with approximately twice the number of reports compared to Saturday, which had the lowest frequency of AE reporting (Fig. 8). The times of day with the most frequent AE reporting were between 7 and 11 am, with another peak observed at 9 pm. Conversely, the least frequent AE reporting during the day occurred between midnight and 5 am (Fig. 9).

Usage rates for questionnaire completion using the KarmApp were in KARISMA 2 68.8%, KARMA Creme 72.4% and KARISMA Endoxifen 97.1% (Table 7). Across the age groups, questionnaire completion using KarmApp was lowest among the oldest (Table 8). Only studying the group aged ≤ 55, showed that KarmApp was used for questionnaire completion frequencies in KARISMA 2 79.2%, KARMA Creme 71.7% and KARISMA Endoxifen 97.1% (Table 9).

**Table 7:** Proportion of participants responding to questionnaires via KarmApp by study

	Yes (%)	No (%)	Total
KARISMA 2^*^	948 (68.8)	430 (31.2)	1378
KARMA Crème^**^	63 (72.4)	24 (27.6)	87
KARISMA Endoxifen^***^	233 (97.1)	7 (2.9)	240

^*^Recruited participants November 2016 to March 2019

^**^Recruited participants August 2018 to October 2018

^***^Recruited participants December 2021 to November 2023

**Table 8:** Proportion of participants responding to questionnaires via KarmApp divided by age group

	Yes (%)	No (%)	Total
40– 49	592 (86.9)	89 (13.1)	681
50– 59	392 (75.8)	125 (24.2)	517
60– 74	260 (51.3)	247 (48.7)	507

**Table 9:** Proportion of participants (age ≤ 55) responding to questionnaires via KarmApp by study

	Yes (%)	No (%)	Total
KARISMA 2	593 (79.2)	156 (20.8)	749
KARMA Creme	33 (71.7)	13 (28.3)	46
KARISMA Endoxifen	233 (97.1)	7 (2.9)	240

**Fig. 10:**
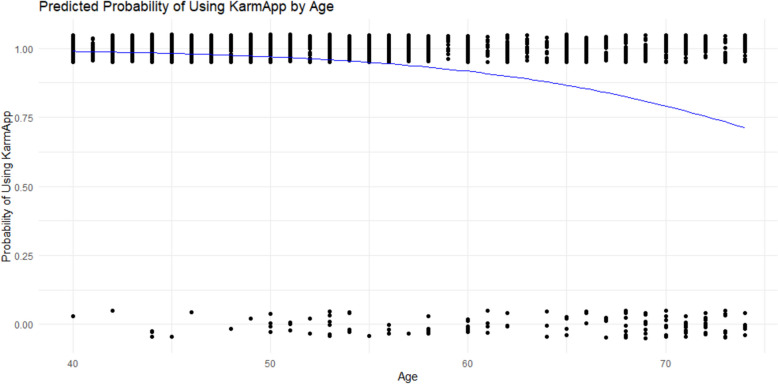
The figure is a plot of the logistic regression analysis of probability of using KarmApp with increasing age, using age as a continuous variable

Further analyzing the age factor’s association with using KarmApp, with logistic regression, gave a coefficient of age = −0.10698, i.e. negative, indicating that as age increases, the likelihood of using the app decreases. This finding was KarmApp is statistically significant (*p* < 0.001). However, as shown in Fig. 10, also in the highest studied ages the proportion using KarmApp was high (around 75%).

## Discussion

We have presented the KarmApp’s development, usage patterns, the impact of age and calendar period on utilization, together with participant experiences and our reflections on implementation and usage. It highlights the importance of choosing a strategy for building a generic tech platform, open to further development and additional features. We acknowledge that there are few reports like this in the literature. There may be an under-reporting from what exists, but it probably also rare that a digital reporting solution is developed by an academic research group.

Our main findings was the increasing usage of the KarmApp over time and that the age factor plays a role. We report a decrease in KarmApp use with increasing age but the use is as high as ≈ 75% among women above70 years.

Of the different functions “Study Activities Overview” was the one most frequently used. This is an example of how the app function as a support for the users and potentially empowering the participants. It helps participants to manage and adhere to their study schedules, including treatment compliance, a critical aspect of clinical trial participation. This is also applicable to any chronic disease management setting. Our data also shows that KarmApp is an attractive and accessible AE reporting tool, compared to the other reporting channels. This is crucial feature for safety and monitoring in clinical trials.

The positive trends in KarmApp usage over time may have several explanations. It can be attributed to the growing use of smartphones and apps among the general public, but also to the continuous optimization of the user-friendliness of KarmApp. Questionnaire completion rates were similar in KARISMA 2 (68.8% of all participants used this function; 79.2% in the 40–55 age group) and KARMA Creme (72.4% of all; 71.7% in the 40–55 age group). Results from the KARISMA Endoxifen indicate an even higher usage rate (97.1%), demonstrating a positive trend also for more complex tasks to be performed using the app **(**Table 9). Analysis of AE reporting patterns unveiled that a substantial portion occurs during the beginning of the week and in the mornings and evenings. This temporal pattern suggests that participants actively use the app to report events at specific times, possibly aligning with their daily routines (Figs. 8 and 9).

Strengths with the study are the relative uniqueness, comprehensiveness of the data and experiences spanning over more than seven years. Collecting participant feedback was useful in the development process.

Weaknesses were the inability to control for confounders like education level and previous digital tool experience of the participants. It could also argued that our data are difficult to generalise given the Swedish context, with a high proportion of smartphone users among the population. In Sweden, smartphone users aged 16–85 years were 7.07 million in 2020 [31], and the number of monthly active smartphone users is projected to reach 9.77 million individuals by 2029, covering nearly the entire Swedish population [32]. Lastly, even though much effort was put into listening to the end-users’ experiences, this could have been done in more formal and structured ways.

Trends in society indicate increased digitalization and the use of mobile technologies. In 2029, there are projected to be 6.38 billion smartphone users worldwide; the current number of smartphone users worldwide is 4.88 billon, accounting for 60.4% of the global population [33]. But there are still some general potential limitations associated with a wide-spread use of mobile health (mHealth) apps, e.g. that not everyone has an adequate mobile device. Even though most citizens in Sweden (92%) owned a mobile device in 2019 [34], with 86% using products that support KarmApp (Android or iPhone), some do not. Globally there are huge differences. Adding smartphone possession as an inclusion criterion could thus lead to an inequality and a selection bias with a risk of skewed results. To address this problem, one possibility is to offer a temporary use of devices to non-smartphone-owning participants. Implementing apps in clinical trials may face various other specific challenges, including development costs, legal issues, and negative attitudes among researchers, healthcare professionals, and study participants [9, 12].

A vast number of apps are available, with thousands of health apps being distributed by Google and Apple. The willingness to use mHealth and share data for health research is high, and has been a priority for the WHO since 2005 [5]. These trends support the exploration and further integration of apps in healthcare and clinical trials.

The development process of KarmApp followed an iterative approach, progressing through repeated cycles of requirements specification, design, implementation, testing, deployment, and post-release monitoring. This approach has ensured continuous refinement, making KarmApp increasingly supportive of our clinical trials. In future studies we plan to evaluate user experience in more formal and structured ways, similar to what was done in 2017 but in a larger scale.

## Conclusions

A vast majority chose to use the KarmApp and reported side effects via the app. More prevalent use was seen among younger participants and use increased over calendar period. Supported by our data, KarmApp exemplifies the potential of using mobile technologies in clinical trials.

## Data Availability

The datasets used and/or analysed during the current study are available from the corresponding author on reasonable request.

## References

[1] Zeng E, He W, Smedby KE, Czene K. Adjuvant Hormone Therapy–Related Hot Flashes Predict Treatment Discontinuation and Worse Breast Cancer Prognosis. J Nat Comprehen Can Netw. 2022;20(6):683-9. 10.6004/jnccn.2021.7116 35385829

[2] He W, Fang F, Varnum C, Eriksson M, Hall P, Czene K. Predictors of discontinuation of adjuvant hormone therapy in patients with breast cancer. J Clin Oncol. 2015;33(20):2262–9. 26033800 10.1200/JCO.2014.59.3673

[3] Greer JA, Amoyal N, Nisotel L, Fishbein JN, MacDonald J, Stagl J, Lennes I, Temel JS, Safren SA, Pirl WF. Systematic Review of Adherence to Oral Antineoplastic Therapies. Oncologist. 2016;21(3):354–76. 26921292 10.1634/theoncologist.2015-0405 PMC4786357

[4] Tabi K, Randhawa AS, Choi F, Mithani Z, Albers F, Schnieder M, Nikoo M, Vigo D, Krausz M. Mobile Apps for Medication Management: Review and Analysis. JMIR Mhealth Uhealth. 2019;7(9):e13608. 10.2196/13608 PMC6786858 31512580

[5] Global diffusion of eHealth: making universal health coverage achievable. Report of the third global survey on eHealth. Geneva: World Health Organization; 2016. Licence: CC BY-NC-SA 3.0 IGO. https://www.who.int/publications/i/item/9789241511780.

[6] Greer JA, Jacobs JM, Pensak N, Nisotel LE, Fishbein JN, MacDonald JJ, Ream ME, Walsh EA, Temel JS, Buzaglo J, Lennes IT, Safren SA, Muzikansky A, Pirl WF. Randomized Trial of a Smartphone Mobile App to Improve Symptoms and Adherence to Oral Therapy for Cancer. Natl Compr Canc Netw. 2020;18(2):133–41. 10.6004/jnccn.2019.7354 32023526

[7] Lazarou I, Krooupa AM, Nikolopoulos S, Apostolidis L, Sarris N, Papadopoulos S, Kompatsiaris I. Cancer Patients’ Perspectives and Requirements of Digital Health Technologies: A Scoping Literature Review. Cancers*.* 2024;16(13):2293. 10.3390/cancers16132293 PMC11240750 39001356

[8] Perry B, Herrington W, Goldsack JC, Grandinetti CA, Vasisht KP, Landray MJ, Bataille L, DiCicco RA, Brandley C, Narayan A, Papadopoulos EJ, Sheth N, Skodacek K, Stem K, Strong TV, Walton MK, Corneli A. Use of Mobile Devices to Measure Outcomes in Clinical Research, 2010–2016: A Systematic Literature Review. Digit Biomark. 2018;2(1):11–30. 29938250 10.1159/000486347 PMC6008882

[9] McKenna KC, Geoghegan C, Swezey T, Perry B, Wood WA, Nido V, Morin SL, Grabert BK, Corneli AL and Hallinan ZP. Investigator Experiences Using Mobile Technologies in Clinical Research: Qualitative Descriptive Study. JMIR Mhealth Uhealth. 2021;9(2):e19242. 10.2196/19242 PMC7910119 33576742

[10] Vogel MM, Combs SE, Kessel KA. mHealth and Application Technology Supporting Clinical Trials: Today’s Limitations and Future Perspective of smartRCTs. Front Oncol*.* 2017;7:37. 10.3389/fonc.2017.00037 PMC5346562 28348978

[11] Shaffer KM, Turner KL, Siwik C, Gonzalez BD, Upasani R, Glazer JV, Ferguson RJ, Joshua C, Low CA. Digital health and telehealth in cancer care: a scoping review of reviews. Lancet Digit Health. 2023;5:e316–7. 37100545 10.1016/S2589-7500(23)00049-3 PMC10124999

[12] Perry B, Geoghegan C, Lin L, McGuire FH, Nido V, Grabert B, Morin SL, Hallinan ZP, Corneli A. Patient preferences for using mobile technologies in clinical trials. Contemp Clin Trials Commun. 2019;15:100399. 10.1016/j.conctc.2019.100399 PMC6610628 31312746

[13] Bäcklund M, Eriksson M, Hammarström M, Thoren L, Bergqvist J, Margolin S, Hellgren R, Wengström Y, Gabrielson M, Czene K, Hall P. Time to Mammographic Density Decrease After Exposure to Tamoxifen. Oncologist. 2022;27(7):601–3. 10.1093/oncolo/oyac104 PMC9256030 35605013

[14] Eriksson M, Eklund M, Borgquist S, Hellgren R, Margolin S, Thoren L, Rosendahl A, Lång K, Tapia J, Bäcklund M, Discacciati A, Crippa A, Gabrielson M, Hammarström M, Wengström Y, Czene K, Hall P. Low-Dose Tamoxifen for Mammographic Density Reduction: A Randomized Controlled Trial. J Clin Oncol. 2021;39(7):1899–908. 33734864 10.1200/JCO.20.02598 PMC8189632

[15] Bäcklund M, Eriksson M, Gabrielson M, Hammarström M, Quay S, Bergqvist J, Hellgren R, Czene K, Hall P. Topical Endoxifen for Mammographic Density Reduction-A Randomized Controlled Trial. Oncologist. 2022;27(7):597–600. 10.1093/oncolo/oyac102 PMC9256025 35604960

[16] Atossa. Atossa Therapeutics Reports Positive KARISMA-Endoxifen Trial Results: ATOS-016R. Available: https://clinicaltrials.gov/search?term=ATOS-016R. Accessed 11 Dec 2024.

[17] KARMA. Karolinska Mammography Project for Risk Prediction of Breast Cancer. Available: https://karmastudy.org/. Accessed 9 Dec 2024.

[18] Gabrielson M, Eriksson M, Hammarström M, Borgquist S, Leifland K, Czene K, Hall P. Cohort Profile: The Karolinska Mammography Project for Risk Prediction of Breast Cancer (KARMA). Int J Epidemiol. 2017;46(6):1740–1. 28180256 10.1093/ije/dyw357 PMC5837703

[19] Shakirat Oluwatosin H. Client-Server Model. IOSR J Com Eng. 2014;6(1):67–70.

[20] Node.js. Node.js JavaScript runtime. Available: https://nodejs.org/en/. Accessed 2 Jun 2023.

[21] Microsoft. Microsoft SQL Server. Available: https://www.microsoft.com/en-us/sql-server. Accessed 2 Jun 2023.

[22] Microsoft. Microsoft Dynamics. Available: https://dynamics.microsoft.com/en-gb/. Accessed 2 Jun 2023.

[23] LIME. Lime CRM. Available: https://www.lime-technologies.com/en/lime-crm/. Accessed 2 Jun 2023.

[24] National Cancer Institute. NCI Guidelines for Investigators: Adverse Event Reporting Requirements for DCTD (CTEP and CIP) INDs and IDEs. 2024. Available: https://ctep.cancer.gov/protocoldevelopment/electronic_applications/docs/aeguidelines.pdf. Accessed 9 Dec 2024.

[25] Hammarström M, Gabrielson M, Crippa A, Discacciati A, Eklund M, Lundholm C, Bäcklund M, Wengström Y, Borgquist S, Bergqvist J, Eriksson M, Tapia J, Czene K, Hall P. Side effects of low-dose tamoxifen: results from a six-armed randomised controlled trial in healthy women. Br J Cancer. 2023;129:61–71. 37149701 10.1038/s41416-023-02293-z PMC10307785

[26] Thomas CG, Jayanthila Devi A. A Study and Overview of the Mobile App Development Industry. Int J Appl Eng Manage Letters. 2021;5(1):115-30.

[27] KarmApp on App Store. Available: https://apps.apple.com/gb/app/karmapp/id1144524729?l=sv. Accessed 9 Dec 2024.

[28] KarmApp on Google Play. Available: https://play.google.com/store/apps/details?id=com.ionicframework.karmapp281209. Accessed 9 Dec 2024.

[29] Bæksted CW, Nissen A, Knoop AS, Pappot H. Patients' experience of communication and handling of symptomatic adverse events in breast cancer patients receiving adjuvant chemotherapy. Res Involv Engage. 2019;5:36. 10.1186/s40900-019-0171-1 PMC6868829 31832240

[30] ClinicalTrials.gov. Stockholm Mammography Risk Stratified Trial (SMART). Available: https://clinicaltrials.gov/study/NCT06270355. Accessed 11 Dec 2024.

[31] SCB Statistics Sweden. Use of smartphones and its apps (number of persons) by smartphones and apps, sex, study domain and year. Available: https://www.statistikdatabasen.scb.se/pxweb/en/ssd/START__LE__LE0108__LE0108J/LE0108T30/. Accessed 10 Dec 2024.

[32] Statista. Number of smartphone users in Sweden 2019–2028. Available: https://www.statista.com/statistics/494638/smartphone-users-in-sweden/. Accessed 16 Dec 2024.

[33] Bankmycell. How many smartphones are in the world? Available: https://www.bankmycell.com/blog/how-many-phones-are-in-the-world. Accessed 9 Dec 2024.

[34] Internetstiftelsen. Svenskarna och Internet. Available: https://svenskarnaochinternet.se/rapporter/svenskarna-och-internet-2019/the-swedes-and-the-internet-2019-summary/. Accessed 16 Dec 2024.

